# Dural Tears and Cerebrospinal Fluid Leak in Osteotomy Surgery for Ankylosing Spondylitis: Incidence, Risk Factors

**DOI:** 10.1111/os.70036

**Published:** 2025-04-03

**Authors:** Yue Huang, Fuze Liu, Yifei Li, Qi Zhu, Hai Wang, Jianguo Zhang

**Affiliations:** ^1^ Department of Orthopedics Peking Union Medical College Hospital, Chinese Academy of Medical Sciences & Peking Union Medical College Beijing China

**Keywords:** ankylosing spondylitis, cerebrospinal fluid leak, dural tears, epidural space, osteotomy surgery

## Abstract

**Objective:**

The osteotomy surgery for ankylosing spondylitis (AS) presents a higher risk of dural injury and cerebrospinal fluid leakage compared to conventional spinal surgical procedures. However, there is currently a lack of systematic summaries in this field. This study aims to present the incidence and risk factors associated with dural tears and cerebrospinal fluid (CSF) leakage during corrective osteotomy procedures for AS with kyphotic deformity.

**Methods:**

A retrospective analysis was conducted on patients diagnosed with AS in our hospital between June 2014 and May 2024 who presented with kyphotic deformity and underwent corrective osteotomy, specifically pedicle subtraction osteotomy (PSO) or Smith‐Petersen osteotomy (SPO). A total of 110 patients were included in this investigation. Among them, 98 patients underwent PSO (69 received single‐segment PSO; 29 received double‐segment PSO), while 12 patients underwent SPO. The mean age of the participants at the time of surgery was 36.25 years (ranging from 21 to 59 years). Of the total cohort, intraoperative dural tears occurred in 37 patients. Radiological parameters—including sagittal vertical axis (SVA), total kyphosis angle, posterior epidural space thickness at the PSO segment, sagittal alignment of the vertebral canal at the PSO segment, Andersson lesions, and dural ossification—were assessed using spine radiographs or computed tomography (CT) scans analyzed via Surgimap software. The continuous variables mentioned above were primarily compared between groups using independent samples *t* test, while categorical variables were mainly analyzed through the chi‐square test or Fisher's exact test for intergroup comparisons. Additionally, binary logistic regression was employed to further validate the risk factors associated with cerebrospinal fluid leakage in patients undergoing PSO osteotomy.

**Results:**

The overall incidence of dural tears was found to be 33.6%. Specifically, the incidence during PSO procedures was recorded at 36.4%, whereas it was only 9.1% for SPO procedures. The upper lumbar PSO is the surgical segment with the highest probability of dural tears during PSO procedures. This study summarizes the imaging characteristics of patients undergoing PSO, revealing that those who experience dural tears and CSF leakage typically present with a smaller thickness of the epidural space at the osteotomy site and a higher prevalence of Andersson lesions and dural ossification. A multiple linear regression model indicates that reduced thickness of the epidural space at the osteotomy site, along with Andersson lesions and dural ossification, are significant risk factors for dural tears and CSF leakage following PSO surgery.

**Conclusion:**

The total accidental dural tears rate in osteotomy surgery for AS is 33.6%. PSO presents a higher risk compared to SPO procedures. Factors such as the thickness of the posterior epidural space at the PSO segment, Anderson lesions, and dural ossification observed in CT scans serve as predictors for dural tears during PSO procedures. A comprehensive preoperative CT imaging assessment can provide valuable guidance regarding the potential occurrence of accidental dural tears and CSF leakage.

## Graphical abstract

The thickness of the posterior epidural space, Anderson lesions, and dural ossification at the pedicle subtraction osteotomy (PSO) segment observed in computed tomography scans serve as predictors for dural tears during PSO procedures in ankylosing spondylitis (AS) patients.

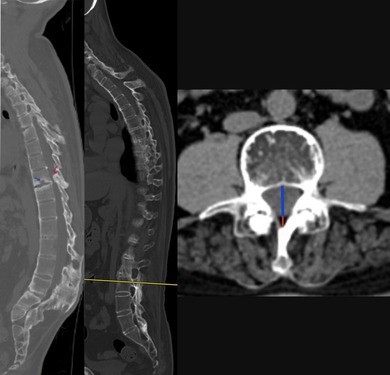

## 1 Introduction

Ankylosing spondylitis (AS) is a chronic inflammatory disease primarily affecting the axial skeleton, particularly the spine and sacroiliac joints [1, 2]. When patients with AS experience spinal involvement, they often develop kyphotic deformities. In cases where severe kyphosis significantly impacts the quality of life or restricts daily activities—such as maintaining a horizontal gaze—surgical intervention becomes an important treatment option for AS [3]. The primary objectives of surgery are to correct the kyphotic deformity and improve patients' ability to maintain a horizontal gaze [4]. Some patients with AS exhibit concurrent involvement of both the spine and hip joints. The treatment sequence for these patients is personalized based on their clinical condition. Traditional perspectives advocate for performing spinal osteotomy first, positing that the hip joint is often misaligned due to a retroverted pelvic position in AS patients. Some emerging academic viewpoints also suggest that prioritizing hip arthroplasty may yield greater clinical benefits for patients with severe spinal deformities or significant hip contractures [5].

Spinal osteotomy techniques play a crucial role during surgical procedures; among them, Smith‐Petersen osteotomy (SPO) and pedicle subtraction osteotomy (PSO) are most commonly employed in patients with AS [6, 7]. Dural tears and CSF leakage are common intraoperative complications associated with spinal surgery [8, 9, 10]. The incidence rates for these complications during lumbar spinal surgeries have been reported to range from 1.8% to 17.4% [11, 12]. The reported incidence of dural tears varies depending on factors such as indications for surgery and the type of osteotomy procedure performed [13]. Previous studies have reported the incidence and risk factors associated with dural tears and cerebrospinal fluid (CSF) leaks during corrective osteotomy for AS with kyphotic deformity. The findings indicated that the overall incidence of dural tears during osteotomy for AS was 17.2%, with patients undergoing PSO exhibiting a higher prevalence of intraoperative dural tears and CSF leakage [14].

Currently, there are no studies addressing the risk factors related to dural tears and CSF leaks specifically during osteotomies performed for AS. This article aims to systematically summarize the following content: (i) the incidence of cerebrospinal fluid leaks and other common complications in patients with AS undergoing osteotomy; (ii) the segmental distribution characteristics of dural tears occurring during PSO osteotomy in patients with AS; (iii) the associated risk factors for dural injuries and CSF leaks in patients with AS undergoing PSO osteotomy.

## 2 Materials and Methods

### 2.1 Participants

A retrospective study was conducted involving patients diagnosed with AS who presented fixed kyphosis deformity and underwent corrective osteotomy between June 2014 and May 2024. A group of surgeons with high surgical volume who underwent the same surgical training completed the operation procedure. This study has been approved by the Peking Union Medical College Hospital Institutional Review Board (K7125).

### 2.2 Inclusion and Exclusion Criteria

The inclusion criteria were as follows: (1) Patients diagnosed with AS combined with kyphotic deformity; (2) Obvious chest or back pain; (3) Complete clinical data; (4) No significant relief of symptoms after conservative treatment. The exclusion criteria were as follows: (1) Unable to tolerate general anesthesia; (2) With hip joint ankylosis, resulting in an inability to cooperate with prone position surgery.

### 2.3 Surgical Process

**FIGURE 1:**
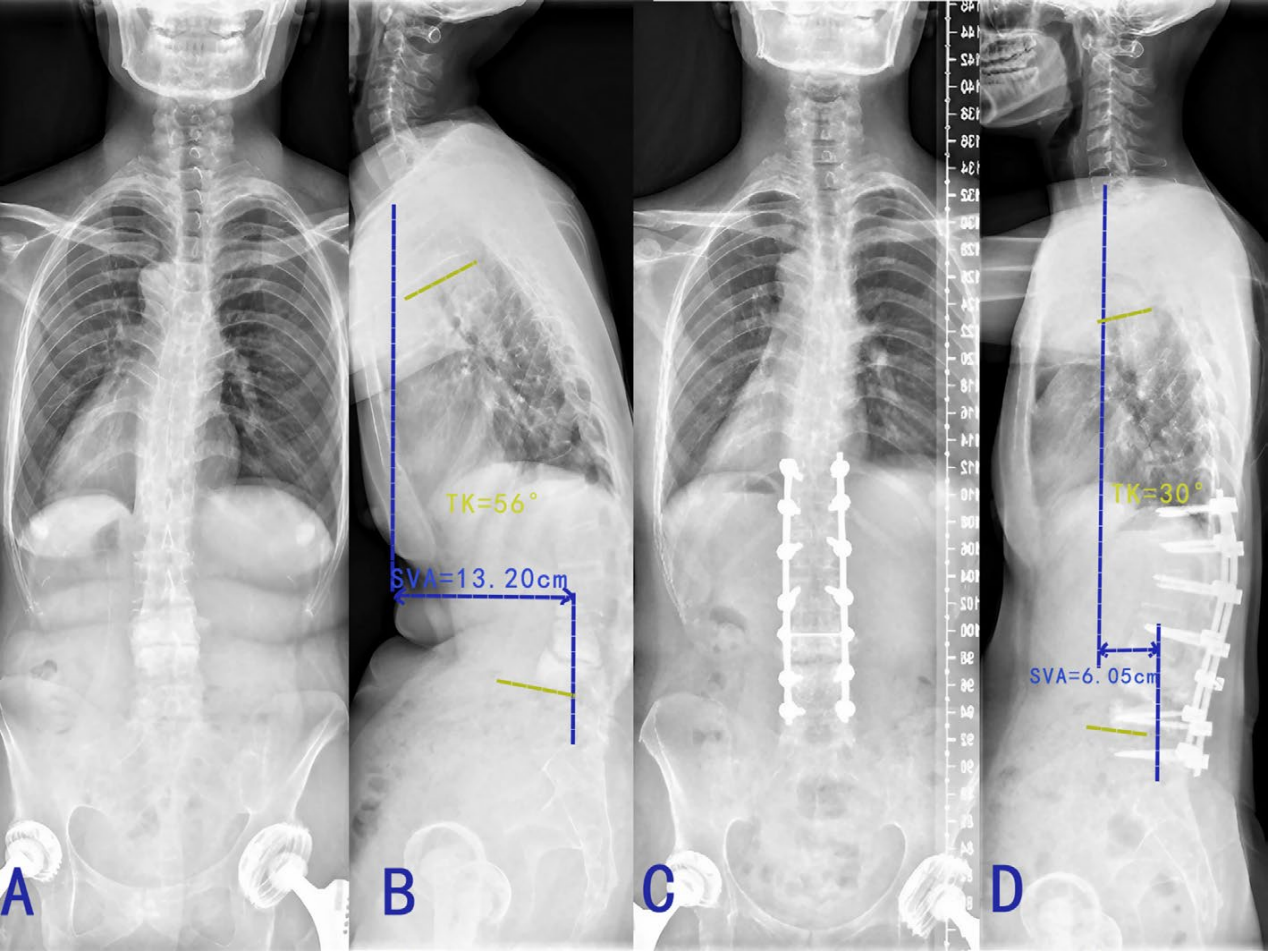
The imaging data of a typical AS patient who underwent Smith‐Petersen osteotomy. (A, B) preoperative standing posteroanterior and lateral whole‐spine radiographs (SVA: 132.0 mm; TK: 56°). (C, D) postoperative standing posteroanterior and lateral whole‐spine radiographs (SVA: 60.5 mm; TK: 30°).

According to the patient's condition, the primary surgical procedures include SPO and PSO. The indications for selection and a general overview of the surgical process are as follows:

The indications for SPOs are as follows: (1) Young patient age (< 50 years) without ossification of the anterior longitudinal ligaments. (2) Absence of osteoporosis, to prevent vertebral body collapse during corrective procedures. In short, following the excision of the spinous processes, implanting robust and reliable pedicle screws [15], then a posterior chevron‐shaped osteotomy measuring 7 to 10 mm was performed, accompanied by bilateral resection of the interlaminar space, ligamentum flavum, and superior and inferior articular processes. The spine was hinged at the posterior annulus fibrosus, opened anteriorly at the still mobile disc space, and shortened posteriorly [16]. The radiological data of a patient who meets the aforementioned surgical indications and underwent SPO osteotomy is presented in Figure 1.

The PSO entails complete removal of posterior elements including laminae, pedicles, and transverse processes. Correction of kyphosis is achieved by gradually straightening the bow‐type frame while closing the osteotomy gap; herein lies an anterior cortex that functions as a hinge. A PSO procedure is indicated for patients with ossification of the anterior longitudinal ligaments or significant osteoporosis. In cases where local kyphosis is severe, PSO osteotomy proves to be more effective. A two‐level PSO is recommended for patients exhibiting severe global kyphosis (GK ≥ 90°), necessitating over 60° of correction to achieve satisfactory postoperative sagittal alignment [17].

The selection criteria for these surgical approaches serve as general principles; however, specific surgical strategies should be tailored based on factors such as the severity of deformity, the characteristics of localized deformities at the site of osteotomy, and patient compliance.

When a dural tear and CSF leakage occurs during surgery, it is currently widely accepted that if conditions permit, an attempt should be made to achieve primary repair [18]. Dural tears are classified into five grades: Grade 1 indicates a tear without defect; Grade 2 denotes a defect smaller than one‐quarter of the circumference; Grade 3 refers to a defect greater than one‐quarter but less than two‐quarters of the circumference; Grade 4 signifies a defect larger than two‐quarters but not completely deficient; and Grade 5 represents complete deficiency [19].

Based on previous literature and clinical experience, this study employs direct suturing for Grade 1 dural defects (only ruptured without loss) using continuous sutures with 6–0 vascular suture material, maintaining an inter‐suture distance of less than 3 mm and a margin of 1 mm. For Grade 2 and 3 dural defects, attempts are made to perform autologous fascia suturing repair while striving to cover the defective area of the dura mater. Due to significant challenges in repairing Grade 4 and above dural defects, no direct repair is attempted in these areas.

In cases where there is tearing of the dura mater during surgery, it is common practice to simultaneously apply absorbable dural patches (Medprin Regenerative Medical Technologies Co.) over the rupture site while utilizing the aforementioned repair techniques. After placing drainage beneath deep fascia layers, meticulous closure of deep fascial tissue is performed. Given that patients with AS present complex scenarios regarding dural tears—such as uncertainty about tear locations or difficulty exposing torn dura due to closed osteotomy gaps—the actual intraoperative management may vary based on specific clinical circumstances. The overarching principle remains focused on achieving primary repair whenever possible, ensuring proper placement of drains, and meticulously closing wounds.

### 2.4 Measurement Parameters

We collected data on demographics, imaging parameters, types of osteotomies performed, as well as details regarding occurrences of CSF leaks. A total of 110 patients (93 males and 17 females) underwent corrective osteotomy; among these, 98 patients received PSO while 12 underwent SPO. The mean age at the time of surgery was 36.25 years (ranging from 21 to 59 years), and the mean duration of disease prior to surgery was recorded as 14.56 years.

**FIGURE 2:**
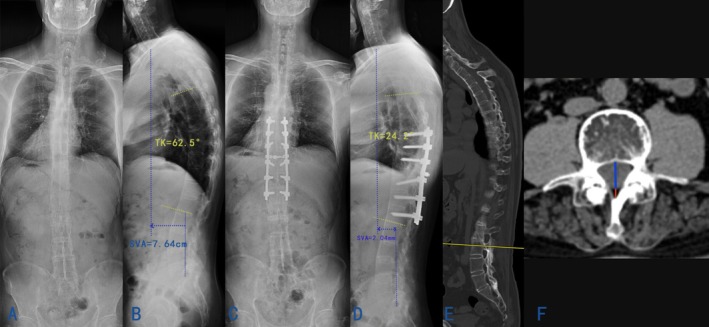
The imaging data of a typical AS patient who underwent single‐segment PSO. (A, B) preoperative standing posteroanterior and lateral whole‐spine radiographs (SVA: 76.4 mm; TK: 62.5°). (C, D) postoperative standing posteroanterior and lateral whole‐spine radiographs (SVA: 20.4 mm; TK: 24.2°). (E) selecting mid‐sagittal images through pedicle roots to corresponding vertebral endplates in CT scan. (F) measuring the distance from the posterior dural boundary to the ligamentum flavum (Red line) in CT scan; measuring the distance from the posterior vertebral edge to the posterior dural boundary in CT scan (Blue line).

**FIGURE 3:**
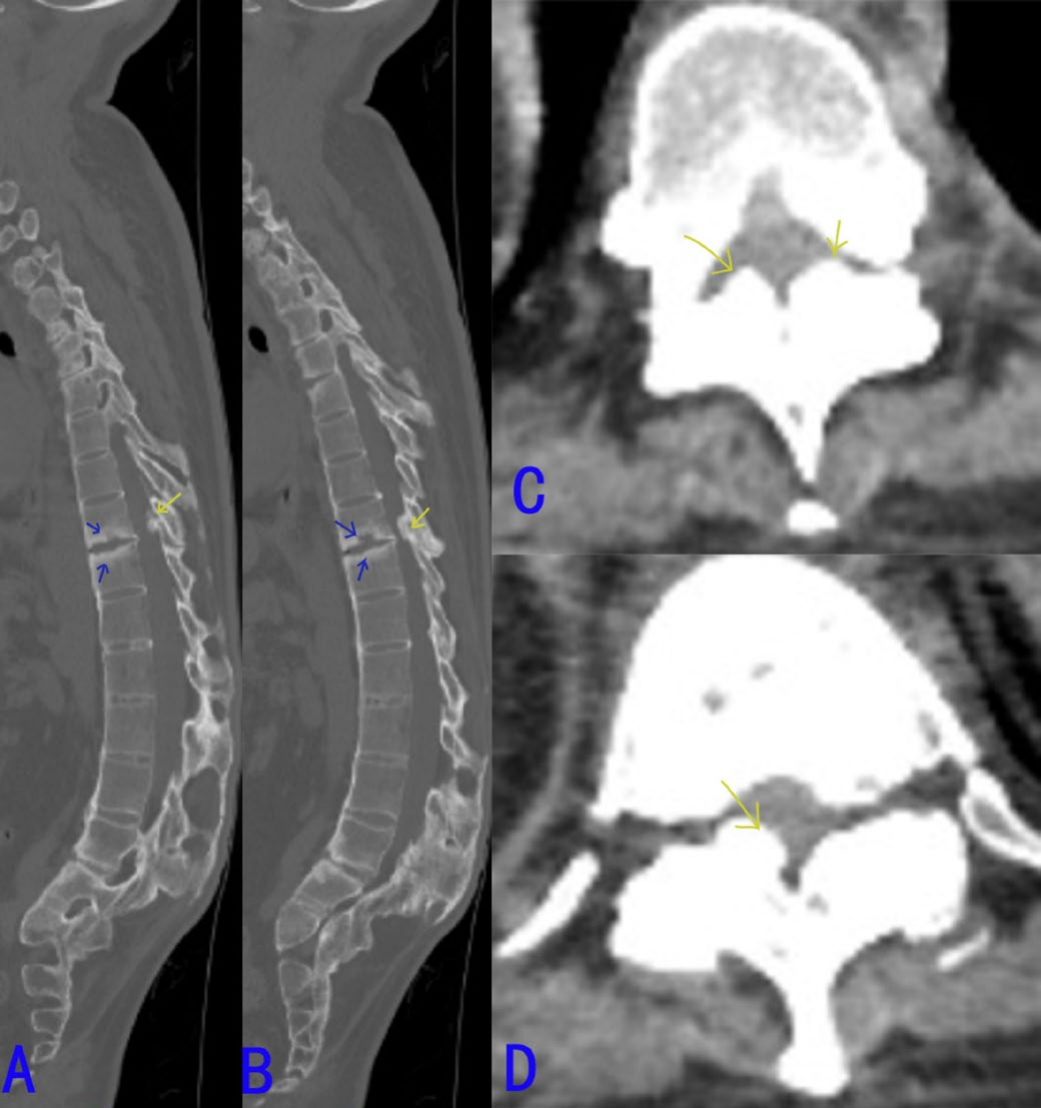
Record the Andersson lesions and dural ossification in PSO osteotomy segment (CT scan). (A, B) The CT scans in various sagittal planes reveal that the patient exhibits Andersson lesions (blue arrow) and dural ossification (yellow arrow). (C, D) The axial CT scans in different planes indicate that the patient has dural ossification (yellow arrow).

All radiological assessments were conducted utilizing our hospital's picture archiving and communication system, focusing on whole‐spine computed tomography (CT) scans performed within one month prior to surgery. Standing posteroanterior and lateral whole‐spine radiographs taken at baseline were independently analyzed by two authors. We recorded various imaging parameters, including the sagittal vertical axis (SVA), Total Kyphosis (defined as the maximum Cobb angle measured from lateral x‐rays, TK), correction degree (the difference between preoperative adjacent vertebral body kyphosis angles at the osteotomy segment versus postoperative angles), as detailed in accompanying Figure 2. We collected specific imaging findings such as Andersson lesions and dural ossification (Figure 3). Epidural space thickness at the osteotomy segment was measured by selecting mid‐sagittal images through pedicle roots parallel to corresponding vertebral endplates on axial CT scans (Figure 2E); this involved measuring the distance from the posterior dural boundary to the ligamentum flavum (Figure 2F). All numerical variables were assessed by two independent spinal surgeons who recorded their average values.

### 2.5 Statistical Analysis

For statistical analysis, SPSS version 17.0 was employed, with a *p*‐value of less than 0.05 considered statistically significant. Data are presented as means ± standard deviations or proportions. The relationships among continuous variables across different groups were evaluated using unpaired samples *t* tests, while categorical variables were analyzed using chi‐squared tests or Fisher's exact test. After conducting preliminary statistical tests, a binary logistic regression analysis was employed to investigate the risk factors associated with dural injury and CSF leakage during the PSO procedure.

## 3 Results

### 3.1 General Demographics Information

A total of 110 patients were included in this study. Among them, 93 patients (84.5%) were male, with an average age of 36.25 years, all undergoing surgical treatment. Within the cohort receiving corrective surgeries via osteotomy, 12 patients underwent spinal osteotomy (SPO), while 69 received single‐segment PSO and 29 underwent double‐segment PSO—specific details can be found in Table 1.

**TABLE 1:** Demographic, surgical, and radiographic data of patients.

Variables	Value
No. of patients (*n*)	110
Male patients, *n* (%)	93 (84.5%)
Age (years)	36.25 (±8.31)
Preoperative height (cm)	161.60 (±12.60)
Preoperative weight (kg)	67.28 (±15.91)
BMI (kg/m^2^)	25.90 (±6.16)
Osteotomy method (*n*)	
SPO	12
Single segmental PSO	69
Double segmental PSO	29
SVA (cm)	
Preop	129.46 (±68.99)
Postop	51.11 (±35.67)
Postop − preop	78.35 (±65.10)
Osteotomy angle (°)	
Preop	63.33 (±20.84)
Postop	32.81 (±14.15)
Postop − preop	30.52 (±23.00)

*Note*: Values are presented as mean ± SD unless stated otherwise.

### 3.2 Summary of Surgical Complications in Ankylosing Spondylitis

Of these 110 patients, intraoperative dural tears occurred in 37 individuals, resulting in an overall incidence rate of 33.6%. This complication is among the most common associated with surgical interventions for AS‐related deformities; detailed complication rates are referenced in Table 2. Notably, within those undergoing PSO procedures, a higher incidence rate of cerebrospinal fluid (CSF) leaks was observed: specifically, among the group receiving single‐segment PSO (*n* = 69), there were reported instances totaling up to 23 cases, leading to an occurrence rate of approximately 33.3%. In contrast, for double‐segment PSOs involving another cohort comprising 29 individuals, there were 13 cases reported yielding an incidence rate of around 44.8%.

**TABLE 2:** Complications within 2 years after operation according to osteotomy type [cases(%)].

Complications	Total (*n* = 110)	SPO (*n* = 12)	Single PSO (*n* = 69)	Double PSO (*n* = 29)
Dural tears and CSF leakage	37 (33.6%)	1 (8.3%)	23 (33.3%)	13 (44.8%)
Neurological deficit	3 (2.7%)	1 (8.3%)	0 (0%)	2 (6.9%)
Coronal imbalance	2 (1.8%)	1 (8.3%)	1 (1.4%)	0 (0%)
Poor wound healing/superficial infection	3 (2.7%)	0 (0%)	2 (2.9%)	1 (3.4%)
Implant failure	2 (1.8%)	0 (0%)	1 (1.4%)	1 (3.4%)
Abdominal wall pain	15 (13.6%)	1 (8.3%)	8 (11.6%)	6 (20.7%)
Digestive complications	11 (10.0%)	1 (8.3%)	7 (10.1%)	3 (10.3%)
Proximal junctional kyphosis (PJK)	1 (0.9%)	0 (0%)	1 (1.4%)	0 (0%)
Pleural effusion	1 (0.9%)	0 (0%)	1 (1.4%)	0 (0%)
Pneumothorax	2 (1.8%)	0 (0%)	0 (0%)	2 (6.9%)
Visual impairment	1 (0.9%)	0 (0%)	0 (0%)	1 (3.4%)

This study involved a postoperative follow‐up period of two years. Due to the nature of the disease, complications such as implant failure and proximal junctional kyphosis (PJK) occurred during the outpatient follow‐up, while other complications were observed during hospitalization. Among the two patients who experienced implant failure, one had a rod breakage at 14 months post‐surgery, and another exhibited pedicle screw loosening at 22 months; both underwent revision surgery. The patient with PJK reported back pain symptoms 20 months after surgery but declined further surgical intervention and opted for conservative treatment. None of these patients with long‐term complications experienced dural tears or perioperative cerebrospinal fluid leakage.

Among the potential complications associated with dural injury and cerebrospinal fluid leakage, three patients presented neurological deficits that coincided with changes in spinal cord monitoring signals during the orthopedic procedures. It is considered that alterations in spinal cord tension during this process may have contributed to neurological impairment. Following postoperative steroid pulse therapy, two out of these three patients achieved complete recovery of lower limb strength, while one patient showed partial recovery.

Of the 37 patients who experienced dural tears and cerebrospinal fluid leaks, 12 reported low‐pressure headaches postoperatively. These symptoms improved significantly through adequate analgesia, supine positioning, and intermittent clamping of drainage tubes when clear drainage was observed (closed for six hours followed by one hour open). Headache symptoms completely resolved following the removal of the drainage tube.

Among three cases presenting poor wound healing or superficial infection, two had concurrent dural injuries and cerebrospinal fluid leaks; their recovery was slower than expected. However, they ultimately achieved wound healing through sufficient drainage and physical therapy interventions.

### 3.3 Characteristics of Osteotomy Segments in Patients With Dural Tears and CSF Leakage

Subsequently, we summarized the segment characteristics associated with CSF leaks in patients undergoing PSO. The statistical results indicated that lower thoracic and lumbar segments exhibited higher rates of CSF leakage; specifically, the leak rates for segments T9 –T12, L1 –L2, and L3 –L4 were found to be 27.6%, 35.4%, and 31.0%, respectively, as detailed in Table 3.

**TABLE 3:** Summary of dural tears and CSF leakage in PSO.

	Total	CSF leakage *n* (%)
Single segmental PSO		
T9–T12	11	4 (36.4%)
L1–L2	35	14 (40.0%)
L3–L4	23	5 (21.7%)
Double segmental PSO		
Lower osteotomy segment		
Thoracic vertebra	1	0 (0%)
L1–L2	9	1 (11.1%)
L3–L4	19	8 (42.1%)
Upper osteotomy segment		
T1–T4	2	0 (0%)
T5–T8	5	0 (0%)
T9–T12	18	4 (22.2%)
L1–L2	4	2 (50.0%)
Summary		
T1–T4	2	0 (0%)
T5–T8	6	0 (0%)
T9–T12	29	8 (27.6%)
L1–L2	48	17 (35.4%)
L3–L4	42	13 (31.0%)

### 3.4 Risk Factors for Dural Tears and CSF Leakage in PSO Procedure

To further investigate the occurrence of dural tears and cerebrospinal fluid leakage, we compared relevant patient demographic data and imaging findings. Due to the lack of a clear clinical consensus regarding dural injury during surgery for AS, our understanding is primarily based on preoperative clinical experience. The experience suggests that such injuries are potentially related to factors such as the degree of deformity, duration of illness, and extent of tissue adhesion. Therefore, in constructing our model, we incorporated typical imaging parameters and objective indicators. Specifically, we included Body Mass Index (BMI) and disease duration as baseline condition factors; SVA and total kyphosis as measures of deformity severity; and posterior epidural space thickness at the PSO segment along with the ratio of posterior epidural space thickness to sagittal alignment of the vertebral canal at the PSO segment as potential quantitative indicators reflecting tissue adhesion severity. Additionally, characteristic lesions associated with AS—Andersson lesions and dural ossification—were also included in our model for analysis.

The analysis revealed that patients experiencing intraoperative CSF leaks exhibited significantly smaller epidural space thicknesses (1.96 ± 0.12 vs. 4.40 ± 0.11, *p* = 0.00) and lower proportions relative to the spinal canal volume (11% vs. 23%, *p* = 0.00); Additionally, the operative osteotomy segments demonstrated a higher prevalence of Andersson lesions (*p* = 0.02) and dural ossification (*p* = 0.03). Detailed information can be found in Table 4.

**TABLE 4:** Risk factors of intraoperative dural tear in PSO.

Factors	Dural tear	Non‐dural tear	*p*
BMI (kg/m^2^)	27.90 (±6.96)	26.20 (±6.71)	0.10
Duration (years)	13.84 (±6.56)	14.53 (±5.96)	0.28
SVA (cm)	144.89 (±73.19)	131.73 (±76.09)	0.18
Total kyphosis (°)	73.32 (±19.86)	65.66 (±21.19)	0.06
Posterior epidural space thickness at PSO segment (mm)	1.96 (±0.75)	4.40 (±1.08)	**0.00**
Posterior epidural space thickness/sagittal alignment of vertebral canal at PSO segment	0.11 (±0.046)	0.23 (±0.056)	**0.00**
Andersson lesion (yes/no)	33/4	8/82	**0.02**
Dural ossification (yes/no)	19/18	0/90	**0.03**

*Note*: Values are presented as mean ± SD for continuous variables and as proportions for categorical variables. Fisher exact test was used for categorical variables. *p* < 0.05 indicates statistical significance. Bold indicates statistically significant *p* values.

Furthermore, multivariable regression analysis with confounder adjustment was conducted on these variables, resulting in indications that posterior epidural space thickness at corresponding osteotomy levels (OR = 0.93, *p* = 0.03), presence of Andersson lesions (OR = 1.50, *p* = 0.00), and dural ossification (OR = 1.40, *p* = 0.00) were identified as independent risk factors for developing CSF leaks in the PSO procedure. Details in Table 5.

**TABLE 5:** Binary logistic regression results of dural tear and CSF leakage in PSO (Method = LR).

Factors	Unadjusted			Adjusted		
	Crude OR	95% CI	*p*	Adjusted OR	95% CI	*p*
Posterior epidural space thickness at PSO segment	0.80	(0.77, 0.83)	**0.00**	0.93	(0.87, 0.99)	**0.03**
Posterior epidural space thickness/sagittal alignment of vertebral canal at PSO segment	0.01	(0.01, 0.03)	**0.00**	0.53	(0.15, 1.84)	0.32
Anderson lesion	2.13	(1.92, 2.38)	**0.00**	1.50	(1.33, 1.70)	**0.00**
Dural Ossification	2.30	(1.94, 2.73)	**0.00**	1.40	(1.22, 1.61)	**0.00**

*Note: p* < 0.05 indicates statistical significance. Bold indicates statistically significant *p* values.

## 4 Discussion

This article systematically summarizes the occurrence of complications such as dural injury and cerebrospinal fluid leakage associated with osteotomy in patients with AS, based on a review of data from our center. The study identifies Andersson lesions, dural ossification, and reduced epidural space thickness as risk factors for dural tear and CSF leakage during PSO in AS operations. These findings provide new insights for the management of surgical complications related to AS in the future.

### 4.1 The Incidence Feature of CSF Leakage in AS Surgery

Incidental durotomy is a prevalent complication encountered during spine surgery, with reported incidence rates ranging from 0.2% to 20% [20]. While dural tears and cerebrospinal fluid leakage are well‐documented potential intraoperative complications of spinal surgery, there remains a paucity of information regarding the true frequency of these occurrences.

Previous studies have indicated that advancing age, revision surgeries, and lumbar stenosis are significantly associated with an increased risk of accidental dural tears, which occur at rates between 0.4% and 15.8% [21]. Current multicenter databases suggest that patients suffering from AS exhibit heightened risks for developing CSF leaks compared to those with other degenerative spinal diseases [22]; however, systematic investigations into specific occurrences related to patients with AS during corrective spinal procedures remain insufficient.

AS is characterized by chronic inflammation leading to joint destruction, synovitis, and inflammation affecting ligaments and tendons [23]. The development of hyperplasia, fibrosis, and adhesions within ligamentous or synovial tissues significantly complicates tissue exposure and dissection during surgical interventions [24]. Consequently, individuals diagnosed with AS face elevated risks for intraoperative CSF leakage when undergoing osteotomies. In our study, we evaluated overall CSF leak rates among AS patients receiving corrective osteotomies at our institution.

We identified direct correlations between the surgical techniques employed—specifically noting increased probabilities associated with PSOs over SPOs—and observed even greater risks in cases involving double‐segment PSOs. Furthermore, instances exhibiting CSF leakage were linked to an increased likelihood of both local and systemic postoperative complications. When such events occur, hospital stays may be prolonged while medical expenses escalate alongside infection risks. A previous case–control study has demonstrated significant increases in venous thromboembolism (VTE), wound complications, meningitis, epidural hematomas, as well as respiratory and gastrointestinal complications among individuals sustaining dural tears [25]. Therefore, when planning surgical approaches, it is imperative not only to ensure effective correction but also to consider the inherent variations across different techniques regarding their respective risks of inducing cerebrospinal fluid leakage. This consideration is essential for tailoring optimal strategies that effectively address individual patient needs while restoring kyphotic deformities and maintaining proper alignment angles.

### 4.2 Characteristics of Osteotomy Segments in PSO With Dural Tears

The incidence of dural injury associated with PSO osteotomy in patients with AS varies across different osteotomy segments. Specifically, the incidence is 0% in the upper and middle thoracic regions, while it ranges from 27.6% to 35.4% in the thoracolumbar and lumbar regions. This discrepancy may primarily be attributed to the limited number of PSO cases involving the upper and middle thoracic segments (*n* = 8), which results in a stronger correlation between incidence rates and individual patient characteristics. A comparative analysis of relevant parameters at various osteotomy sites indicates that although there are some statistical differences in epidural space thickness and local kyphotic angles among different segments, these factors do not appear to have a significant correlation with the incidence of dural injuries observed in this study (Tables S1 and S2). Therefore, regarding the overall differences in dural tears and cerebrospinal fluid leak rates among various osteotomy segments, neither epidural space thickness nor local kyphosis seems to be a primary factor contributing to variations in dural injury rates. It is hypothesized that this phenomenon may stem from the progressive nature of AS, wherein patients typically experience increasing involvement from lower to upper spinal levels. Consequently, inflammatory responses affecting lumbar vertebrae may lead to poorer tissue conditions compared to those seen at thoracic levels, thereby resulting in a higher rate of intraoperative dural injuries.

The present study summarizes both the incidence and distribution characteristics of dural injury during osteotomy procedures for AS and emphasizes the imaging features associated with affected patients. Through CT scans, we confirmed that if the selected osteotomy segment exhibits clear imaging indications of Andersson lesions or dural ossification, the probability of intraoperative dural tears and cerebrospinal fluid leakage significantly increases.

### 4.3 Risk Factors for Dural Tears and CSF Leakage in PSO Procedure

Andersson lesion is a destructive vertebral or disco‐vertebral lesion occurring in the late stages of AS [26], with an incidence rate among patients ranging from approximately 5% to 28% [27]. It is also called spinal pseudarthrosis (SP) [28, 29]. Most of these lesions are seen at the junctional regions, with the thoracolumbar junction being the most commonly involved region [30]. These lesions can manifest as either erosive or sclerotic processes extending into either the disc or adjacent bone [31], and they are typically identifiable through CT or MRI examinations. Various etiologies have been proposed for these lesions, including inflammation, trauma, etc. [32]. Some scholars have reported that Andersson lesion can be categorized into inflammatory Andersson lesion and traumatic Andersson lesion. Inflammatory Andersson lesion represents the natural progression of AS, for which conservative treatment proves effective; conversely, traumatic Andersson lesion constitutes true SP and necessitates surgical intervention [33].

Andersson lesion or SP represents an insidious fracture line that traverses the disc or vertebral body to the posterior column, facilitating abnormal movement. Continuous excessive mechanical tension and anterior component displacement contribute to further bone resorption and hardening reactions, resulting in extensive destructive changes. Pathological studies suggest that the underlying pathological process may involve a reparative mechanism characterized by fibrovascular tissue proliferation originating from the vertebral bone marrow. This fibrovascular tissue also penetrates the vertebral end‐plate and intervertebral spaces, leading to significant disco‐vertebral destruction. The typical pathological alterations include replacement of the interbody disc with fibrous tissue and/or fibrocartilage exhibiting moderate to severe fibrinoid necrosis and cystic degeneration [26]. Therefore, due to the aforementioned repeated organizational repair processes, it can be anticipated that the degree of tissue adhesion in areas affected by Andersson lesions will be relatively severe, which may pose certain challenges during the surgical procedure.

Andersson lesion in AS patients represents an unstable condition that affects all three columns of the spine. The compensatory hyperplasia of osteophytes and surrounding fibrotic tissues often fails to facilitate the proper healing of fractures. Typically, micro‐movements associated with non‐union lead to difficulties in achieving a stable state during the self‐repair process, resulting in a vicious cycle of injury and repair that perpetuates a pathological state within the local tissue structure. Furthermore, these osteophytes and fibrotic tissues may exert varying degrees of compression on the dural sac or nerve roots, leading to significant pain and neurological symptoms [34].

The current surgical treatment for this condition remains a topic of debate. In summary, anterior approaches allow for thorough resection of the lesion and facilitate bone grafting; however, they are associated with a higher incidence of complications related to internal fixation and poor control over kyphotic deformities. Combined anterior–posterior surgeries can enhance the correction of kyphosis and promote bone graft healing, but they come with prolonged operative times and increased risks. Currently, pure posterior fusion is considered a relatively safe, straightforward, and rapid method for treating Andersson lesions. When performed with appropriate control over surgical trauma, it can achieve satisfactory corrective outcomes. Regarding the management of Andersson lesions through posterior surgery, some scholars suggest that osteotomy at the site of pseudoarthrosis may benefit bone graft healing. Conversely, others argue that performing osteotomy at the site of an Andersson lesion carries a high risk of complications and significantly increases surgical difficulty. Some clinical case reports indicate that abnormalities in spinal anatomy may also be associated with Andersson lesions [35]. Therefore, it may be preferable to perform osteotomy in adjacent areas to reconstruct stability at the site of the Andersson lesion using an internal fixation system along with posterior bone grafting. In clinical practice, decisions are made based on various factors including the preferences of the surgical team, characteristics of the deformity, and patients' treatment desires.

In this study, a significant number of patients with Andersson lesions exhibited damage localized to the apex of the kyphotic curve. From the perspective of orthopedic strategies and spinal stability reconstruction, nearly all patients with Andersson lesions underwent osteotomy at this specific site. A small subset of patients (*n* = 2) presented with multiple Andersson lesions, and osteotomy was performed at one of these sites. Notably, none of these patients displayed symptoms indicative of preoperative nerve root dysfunction.

Our research indicates that performing PSO osteotomies in segments exhibiting Andersson lesions in patients with AS is associated with an increased risk of dural tears and cerebrospinal fluid leakage during surgery. This heightened risk may be attributed to tissue damage repair mechanisms and local inflammatory responses, which can lead to the formation of tissue adhesions.

When CT clearly demonstrates dural ossification at a specific site designated for osteotomy, this area typically presents suboptimal tissue conditions; even meticulous dissection cannot ensure separation without risking damage to the dura mater within the epidural space. The relationship between dural ossification and intraoperative adhesion of the dura mater is intricately connected, often resulting in challenges during tissue separation due to factors such as adhesions, which can subsequently compromise the normal structure of the dura. Previous literature has reported on the capacity to predict intraoperative dural ossification and tearing through preoperative imaging studies [36, 37], which aligns with the preliminary conclusions presented in this article. Currently, surgical strategies for osteotomy in AS are increasingly focused on correcting kyphotic deformities and improving sagittal balance, while less attention is given to local imaging characteristics at the osteotomy site and potential treatment difficulties. This study aims to enrich research content in this area. Consequently, when preoperative imaging suggests dural ossification at planned osteotomy levels, it is imperative to anticipate potential risks of unexpected intraoperative dural injuries and adequately inform both patients and their families about these risks. If osseous involvement occupies a significant volume within the spinal canal, consideration should be given to altering the chosen segment for osteotomy to minimize potential intraoperative dural damage.

In our study measuring epidural space thickness at PSO osteotomy levels, we found that patients who experienced intraoperative tears of the dura had significantly smaller epidural spaces compared to those who did not sustain such injuries. The potential underlying cause may lie in the pathological process of AS, a widespread inflammatory condition, where the proliferation and hardening of soft tissues within the spinal canal lead to adhesions between the dura mater and surrounding soft tissues. This is reflected in local imaging findings as a reduction in epidural space thickness. The measurements obtained via CT may serve as an important indirect indicator reflecting local tissue conditions and adhesion status; thus, they could hold clinical value in predicting risks associated with intraoperative dural tears and CSF leakage prior to surgery.

## 5 Strengths and Limitations

This article systematically summarizes the occurrence of accidental dural tears and cerebrospinal fluid leakage complications in patients undergoing osteotomy surgery for AS at our center. Through statistical analysis of imaging parameters, we identified relevant risk factors. Notably, we innovatively propose that a reduction in the thickness of the epidural space observed on CT scans is an independent risk factor for unexpected dural injury and cerebrospinal fluid leakage. This finding provides a reference for preoperative assessment of related complication risks.

The limitations of this study include its nature as a single‐center retrospective investigation with a limited sample size. In this retrospective clinical study, we acknowledge the presence of several potential biases that may impact our findings. Firstly, selection bias may affect the representativeness of the patient population, as our research is confined to those patients who could provide complete medical history information. We have attempted to control this through stringent inclusion and exclusion criteria. Secondly, information bias may arise from reliance on potentially incomplete or inaccurate historical medical records. The primary content in the case data presented herein consists of records documented by resident physicians. For determining complications such as cerebrospinal fluid leaks, we corroborated evidence from multiple sources including medical records and drainage conditions to ensure thorough validation. Imaging parameters were processed by averaging measurements taken multiple times by experienced physicians. We have made efforts to mitigate information bias through these methods. However, despite our best attempts at controlling for confounding factors, confounding bias may still persist since not all potential confounders can be adequately adjusted for in a retrospective design. Additionally, some patients were unable to undergo MRI examinations due to severe kyphotic deformities; therefore, this study primarily relied on data measurements obtained from CT scans. Future experimental designs could incorporate approximated calculations from anatomical data into MRI examination metrics.

## 6 Conclusion

The overall incidence rate of unexpected dural injury during corrective surgeries involving osteotomies in patients with AS was determined to be 33.6%. Notably, those undergoing PSO procedures exhibited a significantly higher rate of intraoperative dural injury compared to those receiving SPO procedures. Preoperative imaging indicating the presence of Andersson lesions, dural ossification, or reduced epidural space thickness correlates with an increased risk for dural tears and CSF leakage during surgery. In summary, preoperative indicators such as Andersson lesions, dural ossification findings on imaging studies, along with diminished epidural space thickness can serve as critical predictors for intraoperative dura tear risk in AS patients undergoing PSO corrective surgeries.

## Author Contributions

Y.H. conducted writing – original draft, review and editing. Y.H. and F.L. conducted data curation and methodology. Y.L. and Q.Z. conducted formal analysis. H.W. and J.Z. conducted project administration, funding acquisition, and supervision. All authors have read and approved the final submitted manuscript.

## Ethics Statement

This study has been approved by the Peking Union Medical College Hospital Institutional Review Board (K7125).

## Consent

Written informed consent was obtained from patients and their parents for publication of this article and any accompanying images. Informed consent was obtained from all individual participants included in the study.

## Conflicts of Interest

The authors declare no conflicts of interest.

## Supporting information


Table S1.

Table S2.
