# Supplementary material for: Dural Tears and Cerebrospinal Fluid Leak in Osteotomy Surgery for Ankylosing Spondylitis: Incidence, Risk Factors
Source: Orthop Surg. 2025 Apr 3;17(6):1669–79. doi: 10.1111/os.70036 (PMC12146137; doi:10.1111/os.70036)
Supplement: Supplementary file 1 — Table S1. [file OS-17-1669-s001.docx]

Table S1. Comparison of epidural space thickness at different osteotomy Segments

|  | T1-T8 (3.81±0.75) | T9-T12 (3.03±1.15) | L1-L2 (3.76±1.62) | L3-L4 (4.05±1.54) |
| --- | --- | --- | --- | --- |
| T1-T8 (3.81±0.75) |  | P=1.00 | P=1.00 | P=1.00 |
| T9-T12 (3.03±1.15) |  |  | P=0.20 | **P=0.03** |
| L1-L2 (3.76±1.62) |  |  |  | P=1.00 |
| L3-L4 (4.05±1.54) |  |  |  |  |

The variables are presented as Mean ± SD, with units measured in millimeters (mm). ANOVA analysis indicates a significant overall intergroup difference, with a p-value of 0.04. Pairwise comparisons were conducted using Bonferroni correction for multiple comparisons. P values that are presented in bold denote statistical significance.
