# Supplementary material for: Dural Tears and Cerebrospinal Fluid Leak in Osteotomy Surgery for Ankylosing Spondylitis: Incidence, Risk Factors
Source: Orthop Surg. 2025 Apr 3;17(6):1669–79. doi: 10.1111/os.70036 (PMC12146137; doi:10.1111/os.70036)
Supplement: Supplementary file 2 — Table S2. [file OS-17-1669-s002.docx]

Table S2. Comparison of local kyphotic angles at different osteotomy segments

|  | T1-T8 (21.88±10.34) | T9-T12 (31.38±10.91) | L1-L2 (25.00±14.90) | L3-L4 (14.98±10.34) |
| --- | --- | --- | --- | --- |
| T1-T8 (21.88±10.34) |  | P=0.35 | P=1.00 | P=0.93 |
| T9-T12 (31.38±10.91) |  |  | P=0.22 | **P=0.00** |
| L1-L2 (25.00±14.90) |  |  |  | **P=0.00** |
| L3-L4 (14.98±10.34) |  |  |  |  |

The variables are reported as Mean±SD, with units in degrees (°). P values presented in bold indicate statistical significance. The ANOVA analysis revealed a significant overall group difference with a p value of less than 0.01. Pairwise comparisons were conducted using Bonferroni correction for multiple comparisons. P values presented in bold indicate statistical significance.
